# Open questions on proteins interacting with nanoclusters

**DOI:** 10.1038/s42004-022-00665-9

**Published:** 2022-04-06

**Authors:** Rodolphe Antoine, Dusica Maysinger, Lucie Sancey, Vlasta Bonačić-Koutecký

**Affiliations:** 1 Institut Lumière Matière UMR 5306, Université Claude Bernard Lyon 1, CNRS, Univ Lyon, F- 69100 Villeurbanne, France; 2 Department of Pharmacology & Therapeutics, McGill University, Montréal, QC H3G 1Y6 Canada; 3 Cancer Targets and Experimental Therapeutics, Institute for Advanced Biosciences (IAB), INSERM-U1209/CNRS-UMR 5309, University of Grenoble Alpes (UGA), 38000 Grenoble, France; 4 Center of Excellence for Science and Technology-Integration of Mediterranean Region (STIM), Faculty of Science, University of Split, 21000 Split, Croatia; 5 Interdisciplinary Center for Advanced Science and Technology (ICAST) at University of Split, 21000 Split, Croatia; 6 Chemistry Department, Humboldt University of Berlin, 12489 Berlin, Germany

**Keywords:** Nanoparticles, Optical materials, Proteins

## Abstract

Interfacing ultrasmall metal nanoclusters (NCs) with proteins can present a dual opportunity: proteins can be used for protecting NCs, and the surface ligands of NCs may interact with proteins. Here, the authors identify and discuss remaining open questions surrounding the bio-NC interface that call for future research efforts.

Ligand protected metal nanoclusters (NCs) have attracted increasing attention due to their fascinating physicochemical properties, in particular luminescence^1^, and their capability to be functional for biomedical applications. Such capability can be achieved by interfacing NCs with biomolecules, in particular proteins (bio-NC interface, Fig. 1a). The bio-NC interface presents a dual role (Fig. 1b, c): on the one hand, proteins can be used for protecting NCs, and on the other hand, surface ligands of NCs may present some interaction (intended or not) with proteins. Here we focus on open questions arising from the interaction of NCs with proteins, considering in vitro and in vivo scenarios and using in silico approaches. In particular, we explore what are the main problems associated with nanocluster functionalization by proteins and what happens within exchange reactions of these proteins with intracellular and extracellular endogenous proteins in living cells? How robust is the bio-NC interface? Could nanoclusters qualify as alternatives or substitutes for some fluorescent dyes in biological sciences?

**Fig. 1:**
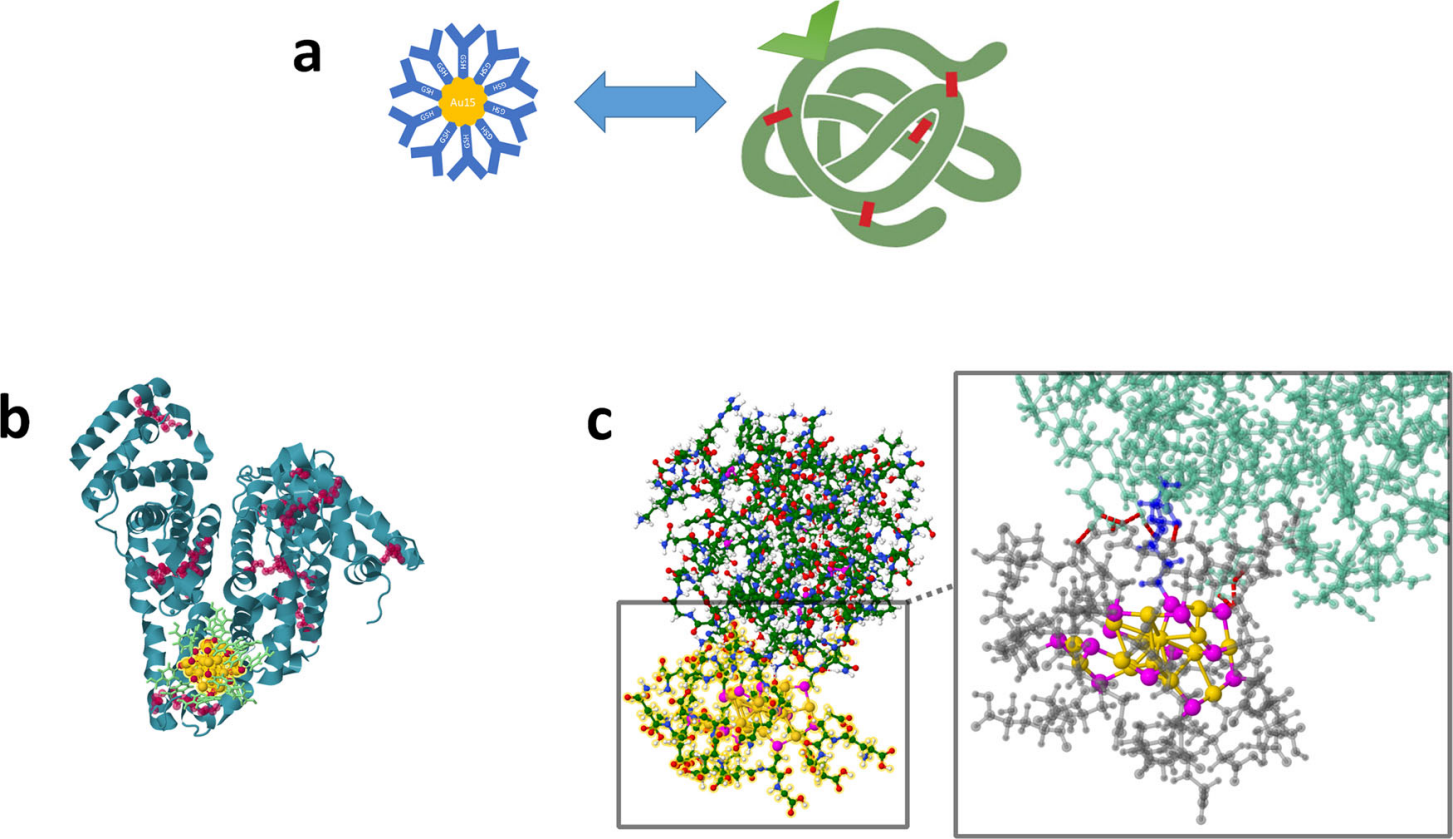
Dual opportunity of the bio-NC interface: proteins can be used for protecting NCs, and the surface ligands of NCs may interact with proteins. **a** Schematics of ligand protected metal NCs and proteins leading to the bio-NC interface. Dual role of the bio-NC interface: **b** proteins can be used as templates for protecting NCs and **c** surface ligands of NCs may be functionalized to present some specific interactions with proteins.

## Proteins interacting with ultrasmall metal NCs

As bare metal clusters are not stable in solution, they need to be protected. The use of proteins as a protective template for metal NC synthesis and functionalization is a rather elegant strategy used in the NC community. The marriage of metal NCs with biomolecules^2,3^, such as proteins, may create some synergistic effects combining the unique optical, electronic, and catalytic properties of the metal NCs with the biological functions of proteins^3,4^. A pioneering work in the field of bio-NC interface was reported by Xie et al. describing an elegant and simple one-pot aqueous synthesis of fluorescent bovine serum albumin–gold (BSA–Au) NCs^4,5^. Following up on this seminal work, a plethora of NCs were synthesized with different proteins for biomedical purposes^4,5^. However, due to the fact that such one-pot synthesis uses proteins both as reductant and stabilizer agents, some alteration of the structure of the protein host and/or uncontrolled formation of clusters in uncontrolled locations inside the protein occurs^6^, limiting the widespread use of such materials in biomedical research. Therefore, new synthetic methods preserving the bioactivity of the protein-directed NCs as well as the structure of the incorporated atomically precise metal cluster is greatly needed. Such synthetic routes could be obtained using ligand exchange to attach a protein molecule onto preformed atomically precise metal NCs. This specific binding was studied mechanistically using a computational approach^7^ and demonstrated experimentally as well as supported theoretically very recently by our groups^8^, where a preformed Au_25_ NC was incorporated in bovine serum albumin (BSA) protein by ligand exchange between the NC surface ligands and BSA cysteine residues (Fig. 1b). This strategy could be further advanced by engineering mutations in proteins with cysteines, allowing for more selective control of the position of atomically precise metal NCs in proteins and of the number of ligands exchanged.

Exploring the dual role of the bio-NC interface will also consist in using “click chemistry” approaches to attach a protein molecule onto an atomically precise metal NC that has well-characterized structural and optical properties. For example, using highly luminescent metal NCs, a protecting ligand could be exchanged for a protein molecule using a targeting linker specifically designed. We developed such a strategy to enhance the detection of protein carbonyls by optical methods. Glutathione (SG)-protected gold NCs (Au_15_SG_13_) were readily functionalized via thiolated aminooxy by a ligand exchange procedure. The as-prepared functionalized Aminooxy-Au_15_ NCs reacted with carbonylated proteins (see Fig. 1c). The targeted carbonylated proteins were then detected by either one- or two-photon fluorescence^9^, and such functionalized NCs could be a useful approach for cellular imaging applications^10^. Other targeting linkers could be coupled through azide-alkyne cycloaddition reactions^11^. The incorporation of a reactive azide group presents challenges for developing functional NCs for applications in glycobiology and analysis of carbohydrate-protein interaction.

## Open questions on proteins interacting with NCs in vitro

**Fig. 2:**
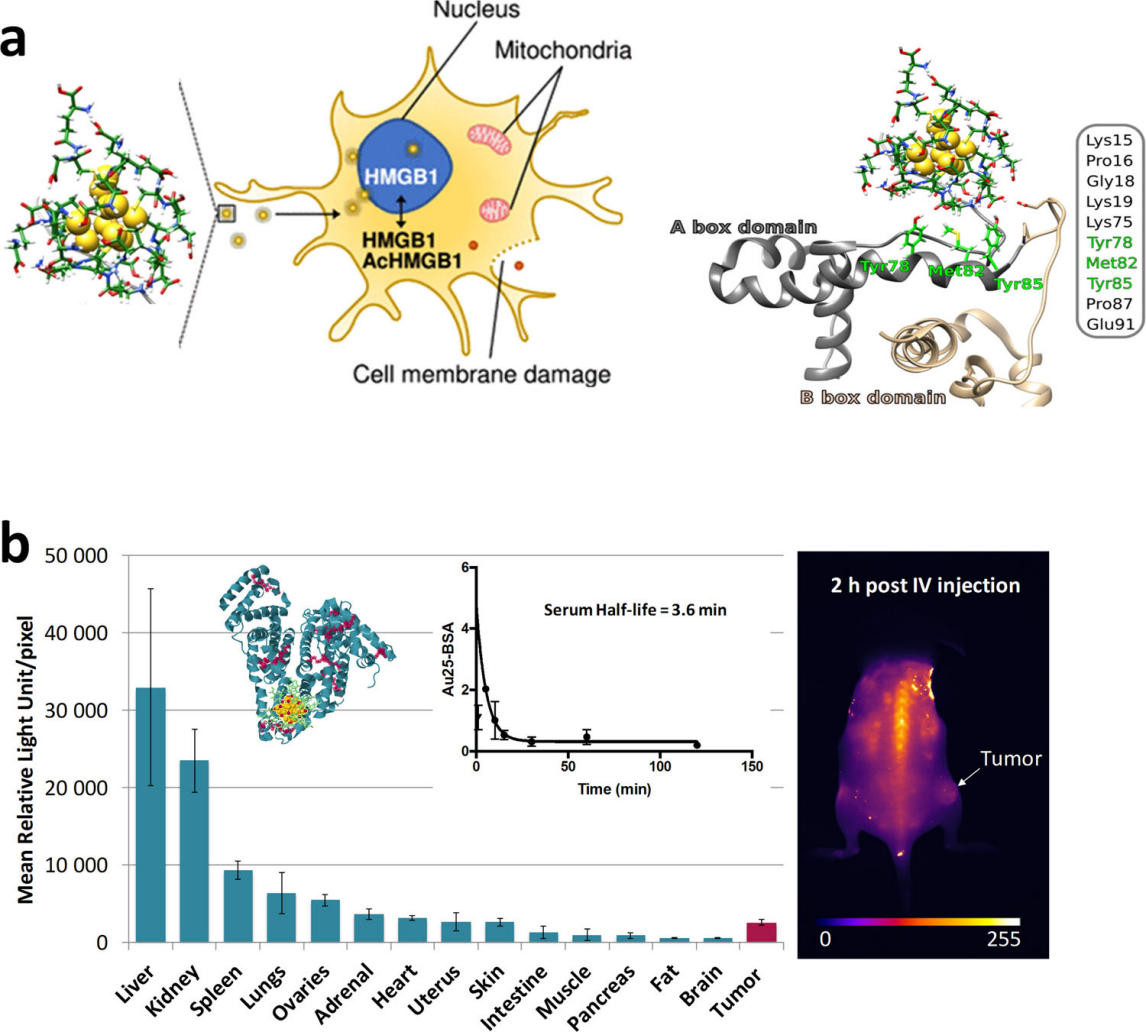
proteins interacting with NCs in vitro and vivo. **a** Illustration of the impact of gold nanoclusters Au_10_SG_10_ on human microglia. Different endpoint parameters used to determine Au_10_SG_10_ toxicity in microglial cells. Reduced form of HMGB1 (redHMGB1: gray ribbon representation for the A box domain of redHMGB1 and the beige ribbon for the B box domain) interacts with glutathione ligands at the surface of Au_10_SG_10_ (stick representation). **b** Au_25_ NCs attached to BSA (illustrated in Fig. 1b) present impressive luminescence properties in SWIR (i.e., emission 1064–1700 nm) enabling in vivo imaging, but extremely short residence time (serum half-life = 3.6 min) and very low cancer targeting potential as displayed with bio-distribution and pharmacokinetics investigations. Error bars represent standard deviations of measurements.

Nowadays, contradictory results exist for cytotoxicity properties of metal NCs. Many factors such as different experimental designs, size and ligands used, different types of tumor cells or untransformed primary cultures may explain such inconsistency. Therefore, the toxicity of NCs both in vitro and in vivo must be assessed before any clinical application should be considered. Despite numerous biological applications of NCs in recent years, knowledge of their interactions within the complex biological environment, in particular the cellular medium, is still limited. For instance, it is still unclear how the surface interactions between cellular proteins and NCs affect the biological activity. The uptake mechanisms of NCs also remain unexplored in a systematic manner. Considering that different cell types are equipped with different surface proteins and membrane properties and that they exist in different states (e.g., physiological, pathological, activated, stimulated, etc.), and considering the complexity of ligand arrangements on nanoclusters of different shapes and sizes, systematic study of uptake mechanisms is an enormous and difficult task. Although many reports have indicated that some NCs have good biocompatibility, a thorough study of the key ingredients affecting the in vitro toxicity merits further investigation. A comprehensive examination of survival and cell death mechanisms needs to be made, particularly in human cells. We recently demonstrated that ligand protected gold (Au) NCs were not inert in human primary astrocytes but revealed organellar reorganization upon AuNC treatment, as well as the activation of key cytoprotective transcription factors. Size and ligand effects are definitely playing a key role^12^. Once again, such an effect might be related to the capability of AuNCs to react with proteins through ligand exchange reactions. Also, similar to larger AuNCs, intracellular reactive oxygen species (ROS) generation by NCs was one of the important contributors to the mechanisms of toxicity^13,14^. However, the advantage of producing nanoclusters with atomic precision allows for unambiguously establishing the links connecting the structure of nanoclusters with their properties upon changes in several biomolecules under oxidative stress, as nicely exemplified with Au_10_SG_10_ (see Fig. 2a). Some of the undesirable effects could be alleviated, at least in part, by scavengers of the ROS and by an increase in the protective ligand stability.

## Open questions on proteins interacting with NCs in vivo

Concerning the practical use of NCs in clinical applications in particular cancer theranostics, there is often a trade-off between residence time in the circulation and cellular uptake^15,16^. Once again, size and ligand influence affect the biodistribution, targeting potential, renal elimination, tumor uptake, and biodegradability of metal NCs. For instance, preformed thiolated protected Au_25_ NCs attached to BSA present impressive luminescence properties in short-wave infrared enabling in vivo imaging (i.e., >1000 nm), but extremely short residence time and very low cancer targeting potential, as commonly observed with gold nanoclusters (Fig. 2b)^15,16^. Each of these parameters must be carefully addressed when designing NCs for specific biomedical applications and their possible clinical translation. There is still room for improving their performance and fate in human tissue, including their circulation in blood and clearance from the body. In particular, we showed that linking AuNCs through the ligand exchange strategy within protein templates can have a strong enhancement effect on their linear and nonlinear optical luminescence properties in the NIR-II region^8^. Longer circulation times and deeper accumulation within the tumor might be achieved by post-functionalization strategies, and ligand protected metal NCs were found to be ideal templates. One of the main advantages of ligand protected NCs is the possibility of creating a NC platform with functional moieties appended onto each surface ligand, dramatically enhancing targeting sensitivity toward cancer materials.

## Open questions on in silico approaches for proteins interacting with NCs

As mentioned, the production of nanoclusters with atomic precision allows for unambiguously establishing the links connecting the structure of nanoclusters with their properties upon changes in several biomolecular environments. Advances in computational chemistry and hybrid calculations of large systems, e.g., with quantum mechanical (density functional)/molecular mechanical QM/MM)^17^ treatment for systems containing one thousand atoms, should also make an impact on understanding the most probable binding sites for NCs within a protein molecule^14^ and the effect of protein residues in controlling NC size and structure (in silico approaches)^8^. Such hybrid methods can address photophysical processes (luminescence in the linear and nonlinear optical regime) in protein-NC systems. A combined experimental and theoretical approach including computational simulation tools can provide a holistic description of the nature of the interactions present at the protein-NC interface. This will allow for the establishment of rules for predicting optimal protein-NC interaction in order to initiate new experiments focused on applications.

The aim which should be realized in the future is the optimization of the efficiency of protein- NC interactions in the context of applications. Present achievements are based on general characterizations of ligated noble metal nanoclusters representing the class of ultrasmall nanomaterials with distinctive structural and optical properties, which have yet to be extended beyond the proof of principle. This means that the optimal size of NCs and the choice of ligands have to be designed for efficient interaction with proteins in the context of improving desirable optical properties, such as high brightness and photo-stability needed for numerous applications in medical diagnostics and medical treatments. The ligands play an additional key role since they participate in drastically enhancing non-linear optical properties (NLO) of liganded NCs in the NIR regime^18^. Moreover, functionalization can be achieved by ligand exchange. An example is functionalized aminooxy Au_15_SG_13_ NCs (obtained by ligand exchange) interacting with carbonylated proteins. Protein carbonylation at the molecular level on model lysozyme was investigated by computational chemistry to determine the nature of binding between the NCs and the protein carbonyls (see Fig. 1c). Altogether, this is proof of principle that functionalized liganded AuNCs can serve for the detection of carbonylation sites and might be more efficient than organic dyes. Such rational design of novel functional thiolate-protected metal NCs with controllable surface chemistry could open new avenues towards many other practical applications, particularly in bioimaging and early medical diagnostics.

## Outlook

The recent studies mentioned above show both the possibility and the need to further investigate the nature and the stability of the bio-NC interface. Advances in electron/X-ray techniques for structural characterization are absolutely mandatory to better understand the protein-NC interactions in complex biological media, but also to probe structural and chemical changes that occur in situ and in real time. Mass spectrometry should also bridge the gap to characterizing increasingly complex ligated metal nanoclusters (using zwitterionic ligands, proteins, multi-shell ligands)^19^. Advancements in in situ spectroscopic measurements will lead to a better understanding of how protein-NCs perform in biological environments and conditions and will help towards improved designs for their intended applications, including the reinforcement of the link between the AuNC and the protein for a longer biological stability.

Super resolution microscopy with life-time imaging and advanced electron microscopy methods allowing for 3D reconstructions from living cells will add to our still limited knowledge of nanocluster fate in diverse cell types and their physiological and pathological conditions. Kinetic parameters showing residence time of nanoclusters in different organelles are currently not available. These would require challenging experimental protocols but they could provide much needed information leading to a better idea about organelle-associated proteins and their roles in interacting with nanoclusters. Human cell organoids are valuable model systems to explore nanoclusters in a more complex cellular environment, complementing those in monolayer cultures and co-cultures.

Computational contributions in establishing rules for designing appropriate interactions between proteins and nanoclusters can contribute to motivate new appropriate experiments. The joint efforts of physical and theoretical chemists, material scientists and biologists are expected to propel protein-protected NCs to ever-greater achievements in biological applications. These joint efforts will contribute in the future to the further development of new nanoclusters with optimized properties.
